# Post-COVID-19 and Irritable Bowel Syndrome: A Literature Review

**DOI:** 10.3390/medicina59111961

**Published:** 2023-11-06

**Authors:** Daniel Paramythiotis, Eleni Karlafti, Matthaios Didagelos, Maria Fafouti, Kalliopi Veroplidou, Adonis A. Protopapas, Georgia Kaiafa, Smaro Netta, Antonios Michalopoulos, Christos Savopoulos

**Affiliations:** 1 First Propaedeutic Department of Surgery, AHEPA University General Hospital, Aristotle University of Thessaloniki, 54636 Thessaloniki, Greece; danosprx@auth.gr (D.P.); kveropli@auth.gr (K.V.); smaronetta2@gmail.com (S.N.); amichal@auth.gr (A.M.); 2 Emergency Department, AHEPA University General Hospital, Aristotle University of Thessaloniki, 54636 Thessaloniki, Greece; linakarlafti@hotmail.com; 3 First Propaedeutic Department of Internal Medicine, AHEPA University General Hospital, Aristotle University of Thessaloniki, 54636 Thessaloniki, Greece; aprotopa@auth.gr (A.A.P.); gdkaiafa@auth.gr (G.K.); chrisavopoulos@gmail.com (C.S.); 4 Intensive Care Unit, AHEPA University General Hospital, Aristotle University of Thessaloniki, 54636 Thessaloniki, Greece

**Keywords:** SARS-CoV-2, COVID-19, post-COVID-19 syndrome, irritable bowel syndrome, gastrointestinal manifestations, disorders of gut–brain interaction

## Abstract

The emergence of post-COVID-19 syndrome (PCS), a complex and multifactorial condition that follows the acute COVID-19 infection, has raised serious concerns within the global medical community. Concurrently, Irritable Bowel Syndrome (IBS), a widespread chronic gastrointestinal (GI) dysfunction, is considered to be one of the most common disorders of gut–brain interaction (DGBI) that significantly affects the quality of life and social functioning of patients. PCS presents a wide range of symptoms and GI manifestations, including IBS. This review aims to analyze the GI involvement and the prolonged symptoms of COVID-19 infection as part of PCS, in order to explore the potential development of post-infection IBS (PI-IBS) in COVID-19 patients. Irritating factors such as enteric infection, psychosocial conditions, food antigens, and antibiotics may lead to abnormalities in the physiological function of the GI system and could be involved in the development of PI-IBS. Through the presentation of the pathophysiological mechanisms and epidemiological studies that assessed the prevalence of IBS as part of PCS, we attempted to provide a better understanding of the long-term consequences of COVID-19 and the pathogenesis of PI-IBS. Even though PI-IBS is becoming a global challenge, there are only a few studies about it and therefore limited knowledge. Currently, the majority of the existing treatment options are referred to non-COVID-19-associated DGBIs. Forthcoming studies may shed light on the mechanisms of PI-IBS that could be targeted for treatment development.

## 1. Introduction

Coronavirus disease-19 (COVID-19) is a systemic infectious disease caused by severe acute respiratory syndrome coronavirus 2 (SARS-CoV-2), which emerged in Wuhan, China in December 2019 and spread worldwide, leading to a pandemic [1,2]. The clinical manifestations of COVID-19 range from asymptomatic to severe or even fatal cases [1,3]. Severe infection is defined by hypoxemia, respiratory insufficiency, and multiple organ failure (MOF) [4]. SARS-CoV-2 is an RNA virus that enters cells via the angiotensin-converting enzyme 2 (ACE2) receptor and a spike protein [5]. ACE2 receptors are mainly expressed in the lung parenchyma, but they are also present in many other cell types, such as in the oral and nasal mucosa, gastrointestinal (GI) tract, pancreas, liver, kidneys, heart, spleen, brain, and the endothelial cells of blood vessels [1,5]. Once SARS-CoV-2 invades these cells, it provokes an inflammatory response that includes the activation of immune cells [1]. In severe cases, the viral infection causes an exaggerated inflammatory response and the release of pro-inflammatory cytokines, leading to hypercytokinemia, also known as a cytokine storm [6].

The majority of COVID-19 patients fully recover. However, a notable number of patients continue to experience a diverse set of symptoms for several weeks or even months after the clearance of the acute infection [1,3]. According to the National Institute for Health and Care Excellence (NICE), the term ‘acute COVID-19’ is used to describe signs and symptoms of COVID-19 that last up to 4 weeks. NICE also defines ‘long COVID’ as the condition wherein signs and symptoms continue or develop after acute COVID-19. This term includes both ongoing symptomatic COVID-19 (persistence of signs and symptoms from 4 to 12 weeks) and post-COVID-19 syndrome (PCS) (persistence of signs and symptoms for 12 weeks or more) [7]. In PCS, symptoms should not be explained by any other diagnosis [7,8].

PCS, also called long COVID, is an emerging, complex, and multifactorial condition, consisting of the residual effects of a previously diagnosed acute COVID-19 infection [9,10]. It may be continuous or recurrent and expressed with one or more persistent symptoms of the acute infection or even with new ones [11]. A study from Italy indicates that, among 143 hospitalized patients, 32% had one or two symptoms 60 days after the onset of the first COVID-19 infection, whereas 55% had three or more [12]. People with PCS are, most of the time, PCR negative, which indicates microbiological recovery. They also present with radiological and biochemical recovery. PCS can be divided into two stages, depending on the duration of symptoms. When they continue for more than 3 weeks but less than 12 weeks, this is called ‘post-acute COVID’. In cases where symptoms extend beyond 12 weeks, it is called ‘chronic COVID’ [11].

The diagnosis of PCS is quite challenging, since the essential time for clinical recovery varies among patients and depends on the severity of the infection. It is worth mentioning that some COVID-19 patients were never tested for COVID-19, so PCS is difficult to diagnose even if they later develop symptoms. Individuals who obtain a negative test, either because the test was performed too early or too late, also add to the diagnostic dilemma [6,11]. Among post-COVID-19 symptoms, GI manifestations have been reported in a notable number of patients [13].

Irritable Bowel Syndrome (IBS) is a chronic GI dysfunction that significantly affects the quality of life and social functioning of patients [14,15,16]. Particularly, according to Zamani et al., high prevalences of anxiety and depression have been found in patients suffering from IBS [17]. It is also considered one of the most common disorders of gut–brain interaction (DGBI) and affects 9–23% of the population globally [18,19]. According to epidemiological studies, women and teenagers are more likely to develop IBS [20].

Patients develop abdominal pain, bloating, and changes in bowel habits, such as abnormalities regarding stool consistency and/or stool frequency [14,16]. IBS often presents with other coexisting conditions, like pain syndromes, an overactive bladder, migraines, visceral sensitivity, and psychiatric conditions [21].

Previous studies have explored the development of IBS after COVID-19 infection. Nevertheless, to the best of our knowledge, a review that gathers all the available information relating to PCS and IBS is absent from the existing literature. This research gap was a motivation to compose the present review, which analyses the GI involvement and the prolonged symptoms of COVID-19 infection as part of PCS and aims to explore the potential development of post-infection IBS (PI-IBS). Hence, we present some brief information about PCS and IBS, including their pathogenesis, symptoms, and risk factors. Additionally, we discuss the GI manifestations of PCS and the factors that could trigger the development of IBS after recovery from COVID-19.

## 2. Materials and Methods

A thorough search was performed in the PubMed library until 24 August 2023, using various combinations of the following keywords: “COVID-19”, “SARS-CoV-2”, “post-COVID-19 syndrome”, “gastrointestinal involvement”, “irritable bowel syndrome”, “symptoms”, and “pathophysiology”. The retrieved articles underwent title screening at first, and then abstract screening to further narrow them down. Irrelevant papers were excluded. Other exclusion criteria included the absence of an abstract, being written in a language other than English, and the inclusion of pediatric patients. The remaining papers were submitted to full-text screening, and those missing data of interest were excluded. The final selection included papers that investigated the relation between PCS and IBS. 

## 3. Discussion

### 3.1. Post-COVID-19 Syndrome

**Figure 1:**
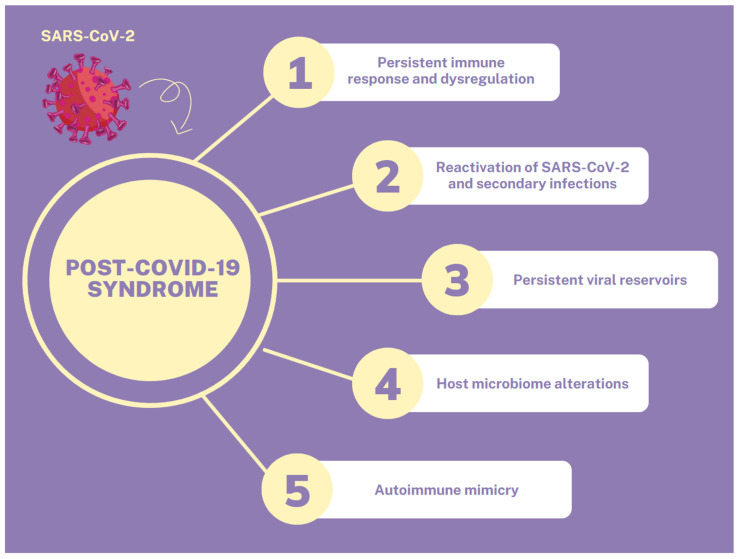
Pathophysiological mechanisms of PCS.

The exaggerated immune response and release of pro-inflammatory cytokines that occurs during infection may induce a long-lasting counterbalancing anti-inflammatory response syndrome (CARS), which causes immunosuppression, in order to maintain immunological homeostasis and prevent MOF. In the case of prolonged immunosuppression, the patient may enter a stage of persistent inflammation, immunosuppression, and catabolism syndrome (PICS), which is one of the potential causes of PCS and also occurs post-sepsis. Moreover, COVID-19-recovered patients are susceptible to relapse or reactivation of SARS-CoV-2 and to secondary bacterial or fungal infections, because of immune dysregulation [6,9]. The persistence of the SARS-CoV-2 infection, with or without symptoms, for up to 3 months, induces long-lasting immune stimulation and the development of PCS. In this way, persistent viral reservoirs could be another potential mechanism for the development of PCS. Other mechanisms that may contribute to the development of PCS include autoimmune mimicry, and the reactivation of pathogens due to host microbiome alterations [6]. All the above mechanisms are summarized in Figure 1.

**Figure 2:**
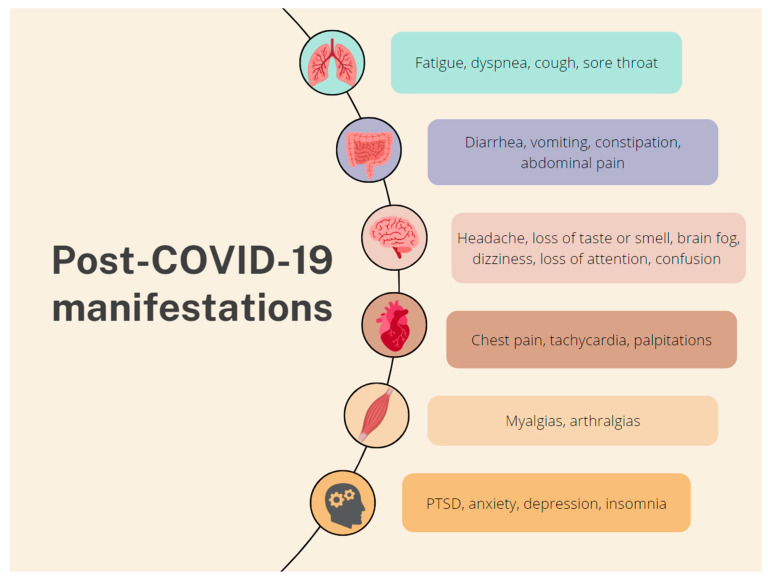
Common PCS manifestations according to the affected system.

Multiple studies have identified numerous COVID-19 manifestations, which affect diverse systems (Figure 2). The main systems are the respiratory (fatigue, dyspnea, cough, and sore throat), the GI (diarrhea, vomiting, constipation, and abdominal pain), the nervous (brain fog, dizziness, the loss of attention, and confusion) and the musculoskeletal (myalgias and arthralgias). Other symptoms include chest pain, tachycardia, palpitations, the loss of taste or smell, and skin rashes. Psychological manifestations, such as post-traumatic stress disorder (PTSD), anxiety, depression, and insomnia, have also been reported [11,22]. However, a follow-up survey conducted by Kirchberger et al. found that fatigue, muscle or joint pain, headache, dyspnea, and concentration problems were the most common persisting symptoms [23]. The prevalence of PCS symptoms seems to be much higher in hospitalized patients compared to those who received treatment for COVID-19 on an outpatient basis [11].

Risk factors that present an association with the development of PCS include sex, age, the number of symptoms that occur during the acute infection, and the presence of co-morbidities [11]. Women present a higher risk compared to men, because of their higher immunological response and hormonal changes. Moreover, COVID-19 patients with a severe initial infection, or those who needed ICU admission and were treated with invasive ventilation, are considered to be a high-risk group for the development of PCS [6].

### 3.2. Gastrointestinal Manifestations of COVID-19

Since the beginning of the COVID-19 pandemic, GI manifestations have been identified among COVID-19 patients with variable incidence (17–53%). Geographical differences may explain some of these disparities [24]. The most frequently occurring symptoms include diarrhea, nausea, vomiting, and abdominal pain [25]. Anorexia may also be present, even though some authors do not consider it a GI symptom [24]. GI symptoms may exist even without any respiratory symptoms or appear after the arise of the latter [21]. SARS-CoV-2 infects and replicates in the GI system, causing the destruction of the intestinal epithelial cells. As a consequence, changes may occur in intestinal permeability, secretion, and malabsorption, which could possibly explain diarrhea and other GI manifestations [24].

Elevated concentrations of aspartate transaminase (AST), alanine transaminase (ALT), alkaline phosphatase (ALP), gamma-glutamyl transferase (GGT), lactate dehydrogenase, and bilirubin have associated SARS-CoV-2 infections with liver damage [2,21]. Elevated serum amylase or lipase in COVID-19 patients is associated with pancreatic injury [5]. Interestingly, a relationship could probably exist between the drugs used to treat COVID-19 infections and GI manifestations [21].

The exact mechanism behind COVID-19-associated GI injury is still unclear and probably multifactorial. Viral replication, systemic inflammatory and immune-mediated effects, ischemia, drug-induced damage, and the exacerbation of other comorbidities may play a key role in GI injury [24]. 

SARS-CoV-2 invades cells via ACE2 receptors, whose genes are highly expressed in the epithelial cells of the small intestine, specifically on the terminal ileum and duodenum, causing local and systemic inflammation [4,21,26]. They are also present in other parts of the GI system, such as the stomach, colon, pancreas, liver, esophagus, and biliary tract [21]. ACE2 is an important regulatory enzyme of the renin–angiotensin system (RAS) and participates both in the systemic and the local RAS [27,28]. The systemic RAS controls the extracellular fluid volume and blood pressure [28]. ACE2 receptors seem to maintain intestinal homeostasis and function by regulating blood flow, increasing the mucosal formation of nitric oxide, and regulating ion transport and paracellular permeability [21]. Furthermore, they stimulate the duodenal secretion of mucosal bicarbonate, the first line of defense against gastric acid, and sodium and water absorption [21,29]. ACE2 may also control amino acid homeostasis and absorption and the formation of antimicrobial peptides and regulate the gut microbiota, independently of the RAS [21,27]. It is worth mentioning that the gut microbiota determines susceptibility to colitis, IBS, and COVID-19 infection. Given that ACE2 contributes to amino acid absorption, it regulates intestinal inflammation too. Hyperactivation of the RAS may be the cause of intestinal inflammation [28]. Much higher fecal cytokines, such as IL-8, have been detected in COVID-19 patients than in uninfected controls, which supports the hypothesis that intestinal inflammation caused by SARS-CoV-2. Also, patients with increased SARS-CoV-2 RNA loads in their stool samples presented with a higher incidence of diarrhea [21].

In the GI tract, ACE2 is essential for the expression of the neutral amino acid transporter B0AT1, and the deprivation of ACE2 may reduce the uptake of certain amino acids, such as tryptophan. Tryptophan seems to be crucial for the immune system, and its diminished uptake from the epithelial cells of the small intestine leads to a decreased release of antimicrobial peptides [28]. According to Hashimoto et al., ACE2 knockout mice challenged with dextran sulfate sodium (DSS) developed alterations in their gut microbiota and severe colitis, in comparison with the wild-type control mice [27]. Thus, the downregulation of ACE2 and its anti-inflammatory activity by SARS-CoV-2 may cause the impairment of bowel physiology, or even MOF, that occurs in some COVID-19 patients [21,28]. The restoration of ACE2 could, therefore, be a sensible therapeutic strategy [28].

### 3.3. Irritable Bowel Syndrome

IBS is considered to be a complicated disease that is affected by various factors with a still unexplored pathophysiology [21,30]. Considering the main symptoms of IBS and more specifically the stool pattern, the syndrome is classified into the following four subcategories: IBS with diarrhea (IBS-D), IBS with constipation (IBS-C), IBS with a mixed pattern (both diarrhea and constipation) (IBS-M), and unclassified IBS, when the stool pattern does not belong in any of the categories above (IBS-U) [20,30,31]. According to Settanni et al., IBS-M and IBS-D are reported to be the most widespread types of IBS [21].

**Figure 3:**
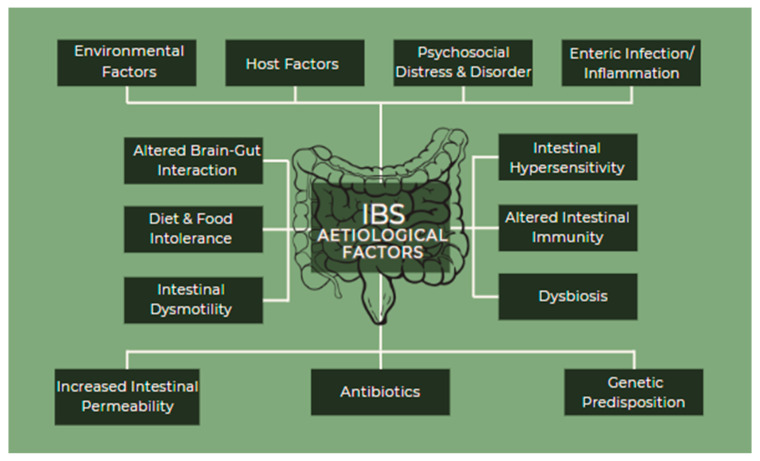
IBS etiological factors.

Despite the fact that the pathogenesis of IBS is not totally understood, there are many etiological factors involved, in various combinations, but not all of them are necessarily present in each patient (Figure 3). They include environmental factors, host factors, psychosocial distress and disorder, enteric infection/inflammation, altered gut–brain interactions, diet and food intolerance, intestinal dysmotility, intestinal hypersensitivity, altered intestinal immunity, dysbiosis, increased intestinal permeability, antibiotics, and genetic predisposition [14,16,30].

The diagnosis of IBS relies on the updated Rome IV criteria [14]. The common characteristic is recurrent abdominal pain for at least 1 day per week, over the last three months, related to, at the minimum, two of the following criteria: (1) related to defecation, (2) associated with a change in the frequency of stool, (3) associated with a change in the form of stool. The criteria should be met in the last three months, with symptoms beginning at least six months before the diagnosis [14,31]. Nevertheless, it is important to mention, that these diagnostic criteria are met in patients with other organic GI diseases and, for that reason, it is quite challenging to make a distinction between the different diseases [31]. Therefore, according to Adriani et al., a beneficial clinical approach should comprise an extensive clinical history (diet, drugs, psychosocial profile, and specific symptoms), a complete physical examination, laboratory tests in patients with typical IBS symptoms (complete blood count, C-reactive protein, fecal calprotectin, and fecal analysis) and diagnostic exams, such as colonoscopy, intestinal ultrasound, and upper GI endoscopy, where appropriate [14].

### 3.4. Gastrointestinal Manifestations of Post-COVID-19 Syndrome

The GI manifestations of PCS are not well recognized, compared to those of the acute infection [32]. According to Batiha et al., the most common GI symptoms include abdominal pain, diarrhea, nausea, vomiting, and a loss of appetite [6]. Some of the studies that have assessed patients with GI symptoms as a part of PCS are presented in Table 1. During acute COVID-19 infection, the increased expression of ACE2 receptors in the epithelial cells of the small intestine may contribute to intestinal infection. Additionally, the persistent shedding of virions from the GI tract may be responsible for some GI manifestations of PCS [32]. SARS-CoV-2 infection may cause alterations in the gut microbiota, with the development of dysbiosis, which can lead to systemic inflammation and pulmonary dysfunction [6]. Other proposed mechanisms support the involvement of cellular damage, enteric nervous system dysfunction, and a prothrombotic state, due to the viral infection. Long-standing GI manifestations could mimic post-infection DGBI, such as IBS. Acute gastroenteritis, which may develop after viral or bacterial infection, seems to be the dominant risk factor for PI-IBS. However, the incidence of PI-IBS following a viral infection has not been studied as much as PI-IBS induced by bacterial infection [10].

### 3.5. COVID-19 and Post-Infection Irritable Bowel Syndrome

Given the high incidence of long-lasting GI symptoms after COVID-19 infection, recent studies have tried to assess the prevalence of newly diagnosed DGBIs, such as IBS, in COVID-19 patients. The first meta-analysis, conducted by Marasco et al., found a frequency of 12% for IBS. The pooled odds ratio (OR), considering only studies with a prospective COVID-19 cohort, was 12.92 (*p* < 0.001 and I^2^ = 0%) [13]. However, in another systematic review of 50 studies, the frequency of IBS after COVID-19 infection was 0.17% (*p* < 0.01 and I^2^ = 96%) [32]. Both studies present several strengths, but also have certain limitations; more specifically, these encompass the inclusion of retrospective internet-based studies, hospitalized patients and outpatients with different clinical courses and follow-up periods, and the subjective nature of patients’ symptoms on follow-up [13,32]. Other studies that attempted to find an association between COVID-19 infection and PI-IBS are summarized in Table 2.

### 3.6. COVID-19 and Post-Infection Irritable Bowel Syndrome Possible Mechanisms

**Figure 4:**
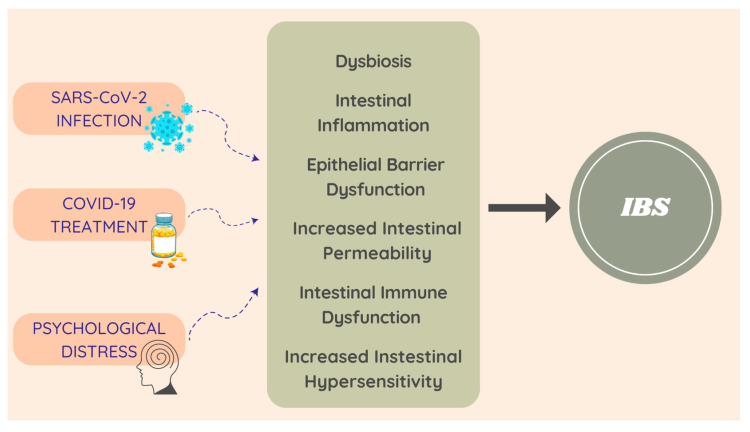
COVID-19 and PI-IBS possible pathophysiological mechanisms.

Irritating factors, such as enteric infection, psychosocial conditions, food antigens, and antibiotics, may lead to abnormalities in the physiological function of the GI system (such as gut dysbiosis, increased intestinal permeability, hyper-sensitivity of the enteric nervous system, immune cell hyper-sensitivity, and dysregulation of the hypothalamic–pituitary–adrenal axis (HPA)) and could be involved in the development of IBS [16,21]. Considering that COVID-19 may affect the normal function of the intestine in different ways, it may be related to IBS pathophysiology, through the following mechanisms (Figure 4) [21,25].

- SARS-CoV-2 Infection

COVID-19 can play a significant role in the pathophysiology of IBS, as it is one of the irritating factors of the gut microbial environment. The bidirectional function of the gut–lung axis, via biochemical and signaling molecules from the immune system, results in disorders from the GI system being frequently related to the respiratory system [21,50]. According to Gu et al., the average community richness and variety of the bacteria was found to be decreased in patients with SARS-CoV-2, when compared to healthy controls (based on the Shannon and Chao diversity index) [21,51]. Moreover, there was a growth in the number of opportunistic pathogens and a reduction in the population of beneficial symbionts [48,50,51].

Interestingly, the malfunction of gut homeostasis may develop in patients with an acute SARS-CoV-2 infection and remain after the clearance of the virus, even in patients without GI manifestations [21,50]. Such an outcome can be explained by the gut–lung axis interaction [21]. Furthermore, both respiratory and GI systems come from the same embryonic organ, namely the foregut, their microbiota grow almost concurrently after birth, and they are affected by common factors [52]. Overall, a disorder of the gut–lung axis, possibly induced by COVID-19, amplifies the manifestation of IBS. It is worth mentioning that there is an increased prevalence of IBS in patients with chronic respiratory diseases, and also that pulmonary manifestations have been reported in about 33% of IBS patients [21,53].

The alterations in the gut microbiome, which are guilty of changing the gut–brain axis, cause functional changes in the GI tract. Consequently, due to the rise in the concentration of calprotectin in the stool and the rise in serotonin levels, there is an important increase in intestinal permeability that is responsible for the development of PI-IBS [21,48].

Secondly, there is another mechanism that is considered to be involved in the enhancement of IBS after infection with SARS-CoV-2. Taking into consideration that SARS-CoV-2 can have an enteric tropism and can cause intestinal inflammation, it is hypothesized that this virus acts with a mechanism similar to that of norovirus [21].

Gastroenteritis is considered a risk factor for inducing PI-IBS and, according to Porter et al., a significant rise in the frequency of functional GI disorders, such as constipation, was found in patients who experienced gastroenteritis during a norovirus infection [21,54]. In addition, according to Marshall et al., there was an increase in the frequency of PI-IBS development in patients with gastroenteritis after infection by food-borne norovirus [21,55]. In spite of the fact that the mechanism through which norovirus causes IBS is unknown, the virus can lead to epithelial barrier dysfunction, increased intestinal permeability, and a mucosal immune response with a high number of cytotoxic intra-epithelial T cells. These intestinal disorders are considered to be etiological factors for IBS development [14,21].

- COVID-19 Treatment

Consequently, there is a non-infective factor that participates in the pathophysiology of IBS by enhancing a dysbiotic condition in the gut. The medications that are used in the treatment of COVID-19 are suspected to play this role [21].

In the first place, broad-spectrum antibiotics (such as azithromycin, vancomycin, and ceftriaxone), which are commonly used in the treatment of SARS-CoV-2 infection, may cause disorders in the composition of the gut microbiome, which may then remain in patients for a long time after the end of the treatment [21,50,56]. They can also induce dysfunction of the intestinal barrier. These changes lead to intestinal infections and intestinal immunity dysfunction, both of which are significant etiological factors for the development of IBS [21].

Antiviral drugs are another option for COVID-19 treatment, especially for the pulmonary phase of the infection. Remdesivir, Lopinavir and Ritonavir are included in this category and are suspected of having an impact on the gut microbiome, causing diarrhea as a side effect, yet there is still limited evidence for this knowledge [21,50].

Hydroxychloroquine has been extensively used in patients for the management of COVID-19 and, according to Balmant et al., it has been related to different degrees of dysbiosis with a dose-dependent effect [21,50,57].

Additional COVID-19 treatment options that are suspected of inducing dysbiosis include corticosteroids and monoclonal antibodies, such as Tocilizumab and non-steroidal anti-inflammatory drugs (NSAIDs). Last but not least, polypharmacy, which is a condition that mainly presents in elderly COVID-19 patients with comorbidities, may also cause an imbalance in the intestinal microbiome [21]. The changes that the above medications cause to the GI system account for the etiological factors that may contribute to IBS occurrence [14,21].

- Psychological Distress

Another possible way through which IBS may develop is stress and the way it activates the HPA axis [21].

COVID-19 is a disease that not only physically afflicts patients, but also has a psychological impact on them, due to various stress-associated factors that were connected to the pandemic the disease caused [21,25,58]. During the acute phase of the SARS-CoV-2 infection, more than 40% of patients reported psychological distress on account of anxiety, fear of isolation, panic, and the thought of a future-threatening disease [21]. Moreover, patients who suffered from COVID-19, especially the hospitalized ones and those with preexisting psychological imbalances, expressed long-term mental effects such as PTSD, irritability, memory impairment, anxiety, insomnia, depression, physical tiredness, and traumatic memories [21,58,59,60].

Either acute or chronically stressful conditions activate the HPA axis, through the discharge of the corticotropin-releasing hormone. This mechanism affects intestinal functions through the regulation of the release of catecholamines, sympathetic and parasympathetic activity stimulation, mucosal immunity, the function of the enteric barrier, the splanchnic blood flow, and the homeostasis of the intestinal microbiome [21]. As a result, the activation of the immune system and gut inflammation may lead to increased intestinal permeability and hypersensitivity, whereas stress-provoked dysbiosis affects the gut–brain axis function [21,25]. 

Lastly, the psychological disorders that patients experience during the COVID-19 pandemic are responsible for changes in their dietary habits, too. Alterations such as a rise in the consumption of simple sugar or carbohydrates, a decrease in water consumption, and a reduction in physical activity have been reported during the pandemic. In addition to dietary changes, eating disorders can be induced by psychosocial stress, or preexisting ones can worsen; these all increase the risk of IBS development [25].

Overall, the COVID-19-induced psychological disorders and their consequences play a role of high importance and can contribute to IBS occurrence [21,25].

### 3.7. Post-COVID Irritable Bowel Syndrome Management

As reported by Cooney and Poullis, even though PI-IBS is becoming a global challenge, there are only a few studies and, therefore, limited knowledge [61]. Physicians should be aware that COVID-19 infection may lead to the development of chronic DGBI, such as IBS. However, up until now, there have been no specific biomarkers or clinical trials testing therapies for GI PCS. The majority of the existing treatment options are referred to non-COVID-19-associated DGBI. Forthcoming studies may shed light on the mechanisms of PI-IBS that could be targeted for treatment development. Consequently, using the already available treatment options for IBS/DGBI, addressing comorbidities, and contributing etiologies, such as autonomic dysfunction or dysgeusia, may potentially improve the patient’s response [25].

The therapeutic strategy of patients with IBS requires a multidisciplinary approach, including the establishment of a strong patient–physician relationship [14,16,31]. IBS management consists of both non-pharmacological and pharmacological therapy and is on an individual basis, depending on the prevalent symptomatology [14,16]. It is worth mentioning that not all patients respond to the same treatment, as non-pharmacological treatment could be effective for some patients, while others may require pharmacological therapy too [16]. Moreover, treatment is directly related to the type of IBS (C, S, M, U), so it is important for patients to be categorized by a physician, in order to determine the most effective therapeutic strategy [16,62]. The following table (Table 3) presents the available treatment options for IBS patients, according to the British Society of Gastroenterology. Treatment should start either with dietary therapy or first-line drugs, depending on the patient’s preference. Second-line drugs are the treatment option in the case of no improvement in symptoms [63]. 

Patients with IBS may benefit from treatment that includes lifestyle alterations, such as stress reduction and regular exercise. Furthermore, there is good evidence that behavioral and psychological treatments directed against IBS symptoms are helpful for many patients, especially those whose symptoms are refractory to drugs [14,20,63]. 

There are multiple guidelines for IBS management, but these significantly conflict with each other [62,63,64]. IBS treatment is changing rapidly as we acquire more knowledge about the disease. Despite the numerous studies that have considered various therapeutic options, there is still no ideal IBS therapy algorithm [16,62,63]. 

## 4. Conclusions

The examination of the potential connection between long-term COVID and IBS reveals an interesting intersection of clinical features, shared symptomatology, and possible underlying mechanisms. COVID-19 can lead to prolonged symptoms beyond the acute phase of infection, especially in what is known as PCS. During the pandemic, it became evident that PCS symptoms, including GI manifestations, are not restricted to those with severe acute COVID-19; rather, they can affect a broad cross-section of patients. Consequently, potential mechanisms that could associate COVID-19 with the development of PI-IBS, have been reported. They include various factors, such as SARS-CoV-2 infection, the impact of COVID-19 treatments, and the role of psychological distress. Additionally, the role of the gut–lung axis, changes in the gut microbiome, and the activation of the HPA axis are highlighted as potential contributors to the pathophysiology of PI-IBS. While the exact nature of the association remains to be fully elucidated, the growing body of evidence emphasizes the need for continued research into the long-term consequences of SARS-CoV-2 infection and the complex interaction between systemic and GI health, including the occurrence of IBS.

## Figures and Tables

**Table 1:** Studies that identified persistent GI symptoms during post-COVID-19 syndrome.

Study Reference	Number of Participants	Hospitalized/ Non-Hospitalized	Type of Study	Assessment Time Since Onset (Average)	GI Symptoms (% of Patients)
Dennis A. et al., 2020 [33]	201	Hospitalized: n = 37; non-hospitalized: n = 164	Cross sectional (analytic)	140 days after the onset	Diarrhea (59.2%)
Tenforde M.W. et al., 2020 [34]	274	Non-hospitalized	Cross sectional (survey)	14–21 days after the onset	Diarrhea (14%), abdominal pain (18%)
Daher A. et al., 2020 [35]	33	Hospitalized	Cohort study	6 weeks post-discharge	Diarrhea (9%)
Valiente-De Santis L. et al., 2020 [36]	82	Non-hospitalized	Observational study	12 weeks after the onset	Diarrhea (1.9%)
Tomasoni D. et al., 2020 [37]	105	Hospitalized	Cross sectional (analytic)	90 days after the onset	GI symptoms (1%)
Eiros R. et al., 2022 [38]	139	Hospitalized: n = 23; non-hospitalized: n = 116	Cross sectional (analytic)	10.4 weeks after the onset	Abdominal pain (4%)
Landi F. et al., 2020 [39]	131	Hospitalized	Cohort study	55.8 days after the onset	Diarrhea (3.8%)
Carvalho-Schneider C. et al., 2020 [40]	150	Hospitalized: n = 53; non-hospitalized: n = 97	Cohort study	30 days after the onset	Digestive disorders (17.3%)
Zhao Y.M. et al., 2020 [41]	55	Hospitalized	Cohort study	3 months after the onset	GI symptoms (30.9%)
Sotiriadou M. et al., 2022 [42]	71	Hospitalized: n = 23; non-hospitalized: n = 48	Observational study	3.12 ± 2.41 months after the onset	Diarrhea (5.6%)
Montoy J.C.C. et al., 2023 [43]	1017	Hospitalized: n = 56; non-hospitalized: n = 943; missing: n = 18	Prospective study	3/6/9/12 months after the onset	GI symptoms (4.8%/1.7%/0.7%/0.3%)
Kirchberger I. et al., 2023 [23]	210	Non-hospitalized	Observational study	2 years after the onset	Diarrhea (18.1%), nausea or vomiting (5.7%)

**Table 2:** Studies that assessed the prevalence of IBS as part of post-COVID-19 syndrome.

Study Reference	COVID-19 Patients/Control Patients n	Hospitalized/ Non-Hospitalized	Type of Study	Follow-Up (Months)	IBS in COVID-19/ Controls n (%)	*p*-Value/ OR	Rome Criteria
Golla R., et al., 2023 [44]	320/320 (A) + 280 (B)	Hospitalized	Cohort study	3 months	8 (2.5%)/0 (0%)	*p* < 0.01	Rome IV
Zhang D., et al., 2023 [45]	190/160	Unknown	Cohort study	6 months	7 (3.7%)/2 (1.3%)	0.189	Rome III
Ghoshal U.C., et al., 2022 [46]	280/264	Unknown	Cohort study	6 months	15 (5.3%)/1(0.4%)	*p* < 0.05	Rome III
Marasco G., et al., 2022 [10]	435/188	Hospitalized	Prospective study	12 months	14 (3.2%)/1 (0.3%)	0.045/10.686	Rome IV
Siyal M., et al., 2023 [47]	303	Hospitalized	Single-center study	6 months	32 (10.6%)	*p* < 0.001/ 0.04	Rome IV
Nazarewska A., et al., 2022 [48]	257	Hospitalized	Single-center study	3 and 6 months	14 (5.4%) and 15 (5.8%)	*p* > 0.05	Rome IV
Farsi F., et al., 2022 [49]	233	Unknown	Cross sectional study	6 months	27 (11.6%)	-	Rome IV

**Table 3:** Treatment of IBS based on IBS subtype.

	IBS-M or IBS-U	IBS-C	IBS-D
**First-line treatments**	* Antispasmodic (e.g., hyoscine, peppermint oil)	* Laxative	* Loperamide
**Second-line treatments**	** Gut-brain neuromodulator (e.g., TCA or SSRI)	* Secretagogue or 5-HT_4_ agonist	* 5-HT_3_ receptor antagonist, eluxadoline, rifaximin
**Psychological therapies**	IBS-specific cognitive behavioral therapy or gut-directed hypnotherapy, if available		

* Continue for 3 months and discontinue if no response. ** TCAs should be the first choice and the treatment should be continued for at least 6 months in case of symptomatic response. TCA, tricyclic antidepressant; SSRI, selective serotonin reuptake inhibitor; 5-HT, 5-hydroxytryptamine; IBS-M, IBS with a mixed pattern; IBS-U, IBS unclassified; IBS-C, IBS with constipation; IBS-D, IBS with diarrhea.

## Data Availability

Not applicable.
